# Acclimation and Characterization of Marine Cyanobacterial Strains *Euryhalinema* and *Desertifilum* for C-Phycocyanin Production

**DOI:** 10.3389/fbioe.2021.752024

**Published:** 2021-11-10

**Authors:** Mahammed Ilyas Khazi, Chenshuo Li, Fakhra Liaqat, Przemyslaw Malec, Jian Li, Pengcheng Fu

**Affiliations:** ^1^ State Key Laboratory of Marine Resource Utilization in South China Sea, Hainan University, Haikou, China; ^2^ Department of Plant Physiology and Biochemistry, Faculty of Biochemistry, Biophysics and Biotechnology, Jagiellonian University, Krakow, Poland; ^3^ School of Biological and Chemical Engineering, Panzhihua University, Panzhihua, China

**Keywords:** *Euryhalinema* sp., *Desertifilum* sp., iron, nitrogen, C-phycocyanin, purification

## Abstract

This study involves evaluation of two native cyanobacterial strains *Euryhalinema* and *Desertifilum* isolated from a mangrove pond in Haikou (China) for their possible phycocyanin (C-PC) production. Maximal growth rate with highest chlorophyll and C-PC accumulation were observed at 28°C and 60 μmol photons m^−2^ s^−1^ photon flux density for *Euryhalinema* sp., while for *Desertifilum* sp. at 32°C and 80 μmol photons m^−2^ s^−1^. Nitrogen and iron concentration trails revealed that double strength concentration of sodium nitrate and ferric ammonium citrate in original BG11 media increased growth rate and accumulation of C-PC for both strains. Three different C-PC extraction methods were tested. The combined extraction protocol of freeze–thaw and ultrasonication markedly increased the C-PC extraction efficiency and attained the food grade purity (*A*_620_/*A*_280_ ratio >0.7), whereas a higher C-PC yield was found with Na-phosphate buffer. Furthermore, the clarified crude extract was used to purify C-PC by fractional ammonium sulfate [(NH₄)₂SO₄] precipitation, Sephadex G-25 gel filtration chromatography, and DEAE-sephadex ion exchange chromatography and attained analytical grade purity (*A*_620_/*A*_280_ ratio >3.9). Taken together, both strains showed their potential to be domesticated for valuable phycocyanin production.

## Introduction

Microalgae are a diverse group of photosynthetic microorganisms that can grow in a wide range of habitats (Khan et al., 2018). They have a high growth rate, simple growth requirements, and can efficiently accumulate large quantities of proteins, lipids, carbohydrates, and carotenoids (Hsieh-Lo et al., 2019). Cyanobacteria, known as blue-green algae, have been gaining considerable attention of various research groups all over the world due to their exceptional carbon fixation potential, high nutritional value, and antioxidant properties. Cyanobacteria are amazing living factories that can be customized for the production of valuable products. Their biomass may be converted to various value-added products such as biofuels, dietary supplements, feed, and cosmetics (Ho et al., 2018; Ma et al., 2020). Moreover, the cyanobacteria species are potential producers of useful secondary metabolites including xanthophylls and phycobiliprotein (Rastogi and Sinha, 2009; Khatoon et al., 2018).

Phycobiliproteins (PBPs) are major light-harvesting cyanobacterial pigments, fluorescent in nature due to the presence of covalently bound, linear tetrapyrrole chromophores called bilins. Cyanobacterial-phycocyanin (C-PC), a water-soluble, nontoxic, fluorescent protein is a part of the PBP pool along with red phycoerythrin (C-PE) and blue green allophycocyanin (C-APC). C-PC is capable of absorbing light at a visible wavelength range (610–620 nm) poorly utilized by chlorophyll (Lee et al., 2017; Khatoon et al., 2018). Major applications of C-PC are as dietary supplement, natural dark blue-colored dye to replace synthetic pigments used in food and cosmetics, fluorescent reagents in clinical or research laboratories, and potential therapeutic agent attributed to its anticancer (Yong et al., 2001), neuroprotective (Min et al., 2015), antioxidant (Sonani et al., 2017), anti-inflammatory (Cherng et al., 2007; Izadi and Fazilati, 2018), and antimicrobial (Shanmugam et al., 2017; Pourhajibagher et al., 2019) properties. Hence, there is great demand of C-PC at a commercial level. According to the reports, in 2016, the market of C-PC as food colorant extended to US$13 billion with nearly 6.5% annual growth rate. The market of C-PC as nutraceutical was assessed between US$6 billion and US$60 billion (Mogany et al., 2018). However, the main challenges in their commercialization and usage in different applications are their low yield during production. Furthermore, the purity of C-PC determines their potential application. For instance, the price of food-grade PC is approximately $0.13 USD per milligram, whereas the price of analytical grade is up to $15.00 USD per milligram (Hsieh-Lo et al., 2019).

Efficient extraction and purification of C-PC from cyanobacteria with enhanced yield is highly essential to expedite its commercialization. To obtain maximum yield of C-PC, the process parameters must be designed to coincide a large amount of biomass production and higher C-PC accumulation in a cell (Lee et al., 2017). The cultivation conditions, including light, temperature, and nutrients, can directly affect the content of C-PC in cyanobacteria. Recent studies have shown that optimal light supply plays a key role in regulating photosynthesis in microalgae and cyanobacteria (Ho et al., 2014; Ho et al., 2018). It is well established that wavelength and intensity of light considerably influence the composition of phycobilisome, and proper adjustment of light can positively affect the energy efficiency as well as C-PC productivity and content (Klepacz-Smółka et al., 2020). Light-dependent variation in the production of PBPs in a cell is known as complementary chromatic adaptation (CCA). There are chances to precisely enhance the accumulation of desired pigment in PBPs by adjusting the wavelength of light (Lee et al., 2017).

Nitrogen (N) is the key nutrient that is mainly used for cell biomass production and protein synthesis. N concentration in the culture medium directly affects cell growth, biomass production, chlorophyll content, and C-PC accumulation. The presence of N plays a vital role in regulating C-PC accumulation. As reported previously, under nitrogen-depleted conditions, C-PC content decreased rapidly, while the biomass increased since PBPs can be utilized by the cell as nitrogen sources (Chen et al., 2013; Khazi et al., 2018). Iron is an imperative microelement for the growth and biological processes of cyanobacteria. It has a significant role in photosynthesis, enzymatic reactions, energy transfer, respiration, synthesis of protein, and nucleic acids. Iron also improves the absorption rate of nutrients, which, in turn, increases the metabolic and photosynthetic activities of cells, leading to a higher growth rate (Mogany et al., 2018; Kumar Saini et al., 2020).

After obtaining maximum production, extraction of C-PC from cyanobacteria is a challenge due to the intracellular location, small cell size, and a thick cell wall (Lee et al., 2017; Khatoon et al., 2018). C-PC yield depends on the extraction method. However, no standard method of extraction can be proposed due to the reason that success of extraction methods depends on the type of cyanobacterial strain as cell wall thickness and size are variable among different cell types. A number of techniques are commonly used to extract C-PC, including chemical, physical, and enzymatic treatments. A combination of two or more methods is often considered as the most efficient C-PC extraction strategy from cyanobacteria (Chittapun et al., 2020). Similarly, for C-PC purification, combinations of various techniques, including fractional precipitation and chromatography methods, are generally employed (Safaei et al., 2019). In general, employing the proper method in both extraction and purification processes is much important to achieve maximum yield and high purity of C-PC.

Above all, one more crucial step for the efficient production of C-PC is the selection of cyanobacterial optimal strain. The effective production and successful application of C-PC depend on the nature of organism, its growth characteristics, ability to scale-up, and, predominantly, accumulation and yield of C-PC (Mogany et al., 2018). Therefore, the discovery of cyanobacteria capable of producing C-PC will remain a priority in biotechnological research. In the present study, two cyanobacterial strains belonging to the genera *Euryhalinema* and *Desertifilum* were isolated from the dynamic and harsh environment of mangroves especially high saline conditions, inconsistent freshwater supply, and low oxygen availability. The main aim of the study was the production of C-PC from these cyanobacterial isolates of extreme environment. To our knowledge, no data is available on the growth and C-PC production of *Euryhalinema* and *Desertifilum* except a conference proceeding reporting C-PC production by *Desertifilum* (Melendez-Antonio et al., 2019). Due to the fact that growth, biochemical composition, and C-PC content of cyanobacteria depend on process parameters and also vary from species to species, the effect of light, temperature, nutrients (nitrogen and iron) on biomass growth, and C-PC production, as well as its extraction and purification became the main subjects of investigation in this study.

## Materials and Methods

### Isolation and Identification

Two cyanobacterial species, CF1 and CF3, were isolated from a mangrove pond located in Hainan University Campus, Haikou, China. Water samples were serially diluted (10^1^–10^5^) in sterile distilled water, and all dilutions were streaked on 1% BG-11 agar plates. The plates were incubated for 2 weeks under a light intensity of 30 μmol photons m^−2^ s^−1^ (16-h light/8-h dark photoperiod) at 25 ± 1°C. After the incubation, the morphologically distinct colonies were picked and purified by repeated streaking method on fresh BG-11 agar plates followed by incubation for 2 weeks under the same conditions (Rippka et al., 1979). Colonies from pure cultures were serially diluted and observed under a microscope using bright field (Nikon ECLIPSE Ci-S) with ×40 for morphological characterization. The pure cultures were then preserved in BG11 agar slants at 4°C. The isolated species were identified morphologically according to Chakraborty et al. (2019) and Desikachary (1959). Strains were subsequently cultured in BG11 medium and incubated at 25 ± 1°C under 60 μmol photons m^−2^ s^−1^ light intensity (daylight fluorescent lamps). The cultures were shaken two times daily to avoid clumping.

### Molecular Analysis

Genomic DNA of isolates was extracted using Plant Genomic DNA kit (Tiangen) following the instruction of the manufacturer. Amplification was performed in 20-µl reactions using primers F (5′-CCT​ACG​GGA​GGC​AGC​AG-3′) and R (5′-ATT​ACC​GCG​GCT​GCT​GG-3′). The PCR reactions were carried out using 2xTaq PCR MasterMix II kit (Tiangen), at the following conditions: preheating at 94°C for 5 min, 32 cycles of denaturation at 94°C for 30 s, annealing at 55°C for 30 s, and extension at 72°C for 2 min, followed by another 5 min extension at 72°C. PCR products were analyzed by gel electrophoresis (1.5% agarose gel). Resulting products were then sequenced by Beijing Genomics Institution (BGI) China. The NCBI-BLAST program (http://www.ncbi.nlm.nih.gov/BLAST) was used to compare obtained sequences with GenBank nucleotide sequences of some known cyanobacteria in order to find the closest-related organisms.

### Evaluation of Light, Temperature, and Nutrients (Nitrogen and Iron) on Growth and Phycobiliproteins

To evaluate the effect of different temperatures on two native cyanobacterial species, the cultures were incubated at 80 μmol photons m^−2^ s^−1^ and the temperatures of 24, 28, 32, and 36 ± 1°C, whereas in the light intensity trial, cultures were exposed to different photon flux densities of 40, 60, 80, 100, 120, 150, and 200 μmol photons m^−2^ s^−1^, and the temperature of 28 ± 1 and 32 ± 1°C (optimum values obtained in temperature experiment) for the strains CF1 and CF3, respectively.

First, the experiments were performed using sodium nitrate (NaNO_3_) as a nitrogen source in different concentrations (2, 3, and 4 g L^−1^), and after determining optimum concentration of NaNO_3_, various concentrations of ferric ammonium citrate (C_6_H_8_O_7_.Fe.NH_3_) (12, 18, and 24 mg L^−1^) were tested as iron source. Standard BG11 media were used as a control (1.5 g L^−1^ of NaNO_3_ and 6 mg L^−1^ of ferric ammonium citrate). A set of three 1-L sterile bottles containing 0.8 L of respective medium were inoculated with 8% (v/v) inoculum (homogenous algal suspension) and cultivated under 60 and 80 μmol photons m^−^2 s^−1^ light intensity (optimum values obtained in light intensity experiment) and the temperature of 28 ± 1 and 32 ± 1°C for the strains CF1 and CF3, respectively. Cultures were aerated continuously using filtered air at a flow rate of 2 L min^−1^. To monitor the growth and pigment content, the cultures were sampled at 2-days intervals under aseptic conditions over a period of 16 days.

### Evaluation of Extraction Method for Cyanobacterial Phycocyanin

Biomass, 50 mg [wet, lyophilized, and oven dried (at 50°C, for 5 h)] was mixed with 5 ml of extraction solution CaCl_2_ (1.5%), Na-phosphate buffer (100 mM, pH 7.0), and NH_4_Cl (0.05 M) and soaked for 60 min (Manirafasha et al., 2017; Khazi et al., 2018). Then the mixture was disrupted using the following different methods.

#### Ultrasonication

The ultrasonic disruption of cells was performed using an ultrasonic homogenizer (SCIENTZ-950E CHINA). Biomass suspended in extraction solution was sonicated for 2 min at 20 kHz. During cell disruption, the samples were kept in ice to prevent overheating.

#### Freeze and Thaw

The biomass suspended in extraction solution was frozen at −20°C for 2 h and then thawed at 27 ± 2°C. The freeze–thaw processes were repeated for two cycles.

#### Freeze and Thaw Combined With Ultrasonication

The biomass suspended in extraction solution was sonicated for 2 min at 20 kHz, then suspension was subjected to one freezing (−20°C, for 2 h) and thawing (27 ± 2°C) cycle.

### Cynobacterial Phycocyanin Purification

The C-PC purification was done using (NH₄)₂SO₄ fractional precipitation combined with chromatographic techniques as described previously (Khazi et al., 2018).

### Biomass and Growth Measurement

The 5-ml cell suspension was filtered through pre-dried and pre-weighed PTEF microfiber filters (JIN TENG CHINA) and washed with distilled water. Dry cell weights (DCW) were calculated in g L^−1^ after the membrane was dried at 75°C to a constant weight.

The growth was estimated by measuring chlorophyll a (Chl-a). To determine the cellular content of Chl-a, the culture was centrifuged, and the pellet was suspended in methanol (100%) for 20 min in a water bath at 65°C, followed by centrifugation at 4,500 × *g* for 5 min. The optical absorption of the supernatant was determined at 665 and 750 nm. The Chl-a concentration was calculated with the following Equation 1 (Demirel et al., 2015).

$$\mathrm{Ch}1\mathrm{-a}\left(\mathrm{mg}\;L^{-1}\right)=13.9\left(A_{665}-A_{750}\right)$$ (1)

The specific growth rate (µ) and doubling time (t_d_) were determined at the exponential growth phase of cultures through Chl-a concentration and time (Li et al., 2014) according to the following Equations 2, 3, respectively:

$$\mathrm{Specific}\;\mathrm{growth}\;\mathrm{rate}\;µ=\frac{\left(lnX_{2}-lnX_{1}\right)}{t_{2}-t_{1}}$$ (2)

where *X*_2_ represents the Chl-a concentration (mg L^−1^) at time *t*_2_ (d), and *X*_1_ represents the initial concentration (mg L^−1^) at time *t*_1_ (d).

$$t_{d}=1n2/\mu$$ (3)

### Measurement of Phycobiliprotein Content

To analyze the PBP in the evaluation of temperature, light, and nutrient experiments, the culture was centrifuged and then washed with dH_2_O. The pellet was suspended in 100 mM sodium-phosphate buffer (pH 7.0), and the suspension was sonicated at a frequency of 20 kHz for 2 min followed by centrifugation for 5 min at 4,500 × g.

The C-PC, C-APC, and C-PE contents (mg ml^−1^) were calculated from the optical absorption at 562 nm (*A*_562_), 652 nm (*A*_652_), and 615 nm (*A*_615_), according to the following Equations 4–6 (Bennett and Bogorad, 1973). The C-PC yield and total PBP content was calculated as shown in Equations 7, 8.

$$\mathrm{C-PC}\left(\mathrm{mg}\;mL^{-1}\right)=\frac{\left\{A_{615}-\;\left(0.474\;A_{652}\right)\right\}}{5.34}$$ (4)

$$\mathrm{C-ΑPC}\left(\mathrm{mg}\;mL^{-1}\right)=\frac{\left\{A_{652}-\;\left(0.208\;A_{615}\right)\right\}}{5.09}$$ (5)

$$\mathrm{C-PE}\left(\mathrm{mg}\;mL^{-1}\right)=\frac{\left\{A_{562}-\left(2.41\;C-PC\right)-\;\left(0.849\;C-APC\right)\right\}}{9.62}$$ (6)

$$\mathrm{yield}\left(\mathrm{mg}\;g^{-1}\right)=\frac{\left(C-PC\right)V}{DB}$$ (7)

Total PBP=(C−PC)+(C−ΑPC)+(C−PE) (8)

Yield is the extraction yield of C-PC in mg of C-PC/dry biomass (g), C-PC is C-phycocyanin content (mg ml^−1^), V is the solvent volume (ml), and DB is the dried biomass (g).

### Statistical Analyses

Experiments were performed in triplicate and the data were presented as means and their standard deviations. Data were analyzed by applying one-way analysis of variance (ANOVA); the significant differences among experiments were determined using Tukey’s test at 95% confidence level (SPSS trail version).

## Result

### Strain Characterization

**FIGURE 1:**
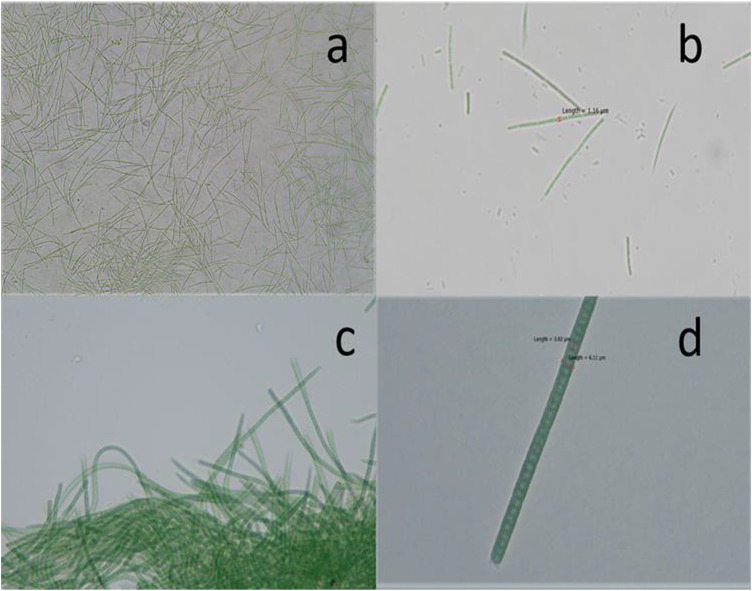
Photomicrographs of (**A, B**) *Euryhalinema* sp. (**C, D**) *Desertifilum* sp.

In the present study, two different isolates were obtained from the water samples collected from a mangrove pond located in Hainan University Campus, Haikou, Hainan Province, China. The morphological analysis of cyanobacteria by bright-field microscopy showed that the isolates CF1 and CF3 belong to the genera *Euryhalinema* and *Desertifilum*, respectively. The cells of CF1 were very thin, filamentous, with filaments typically unbranched, bluish green without any granulated appearance, 1.16 μm broad, thin layer of sheath, and mucilaginous sheath completely absent, no heterocytes and akinetes, flattened ends (Figures 1A,B). The cells of isolate CF3 were bright bluish green, unbranched, straight to flexuous, either solitary or entangled filaments, trichomes 3.02–6.1 μm broad, apical cells conical-rounded and filaments motile (Figures 1C,D). Furthermore, the molecular identification was carried out by partial 16 rRNA gene sequences, and the obtained sequence was compared with the existing sequences in the NCBI database by the BLASTn. The BLAST analysis of the corresponding sequences showed that the isolate CF1 was closely related to *Euryhalinema mangrovii* AP9F (MK402979.1) with sequence similarity of 99.51%, whereas, the isolate CF3 was found to have 99.44% similarity with *Desertifilum* sp. *strain* sp. *EAZ03* (MN255843.1) in the database. The sequence was submitted to the GenBank, NCBI, and -accession numbers (MN855348.1 and MN995387.1) were obtained.

### Influence of Temperature and Light

**FIGURE 2:**
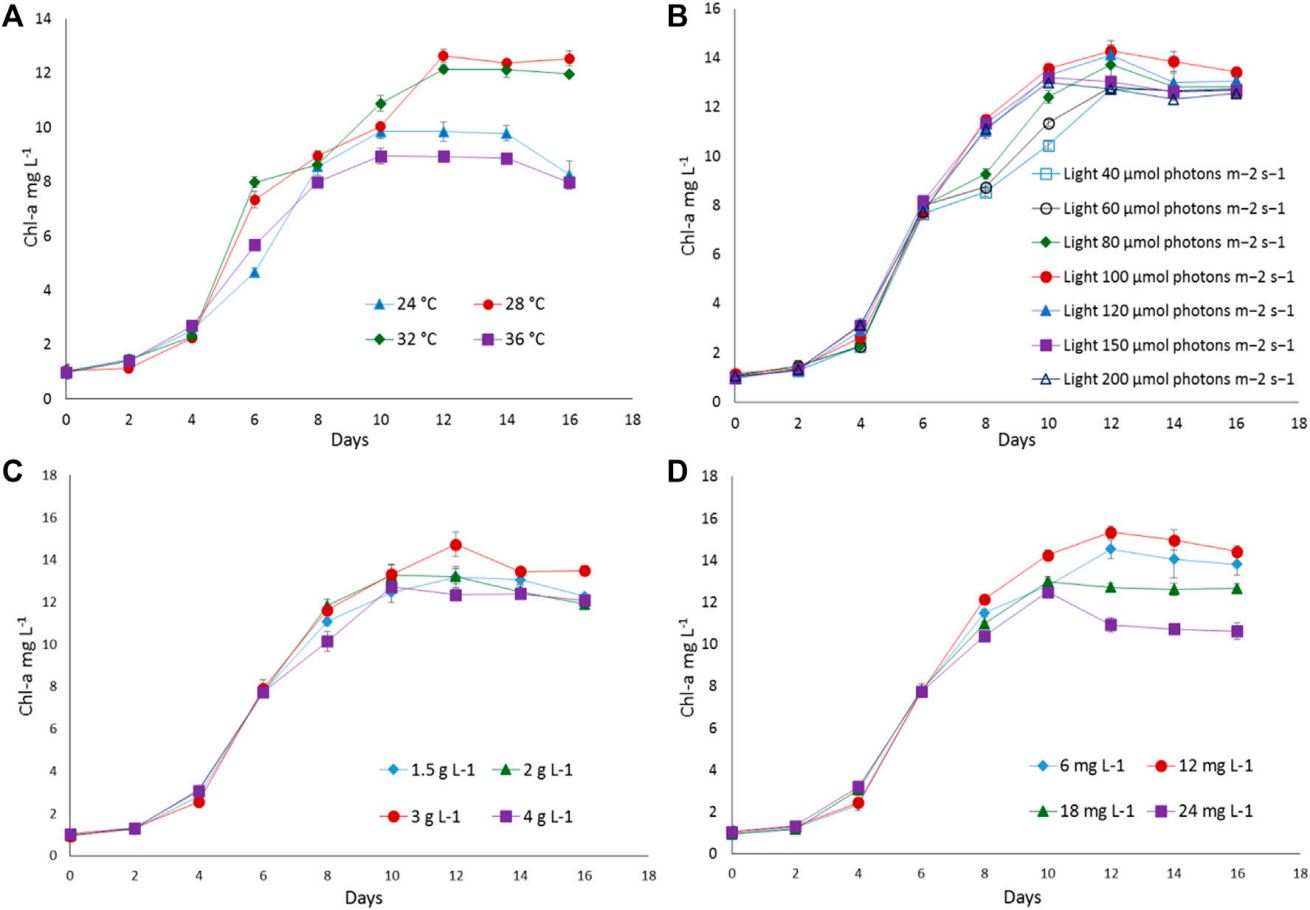
The growth response of *Euryhalinema* sp. to different **(A)** temperatures, **(B)** light intensities, **(C)** sodium nitrate concentrations, and **(D)** ferric ammonium citrate concentrations. Error bars represent ±SD (n = 3).

**FIGURE 3:**
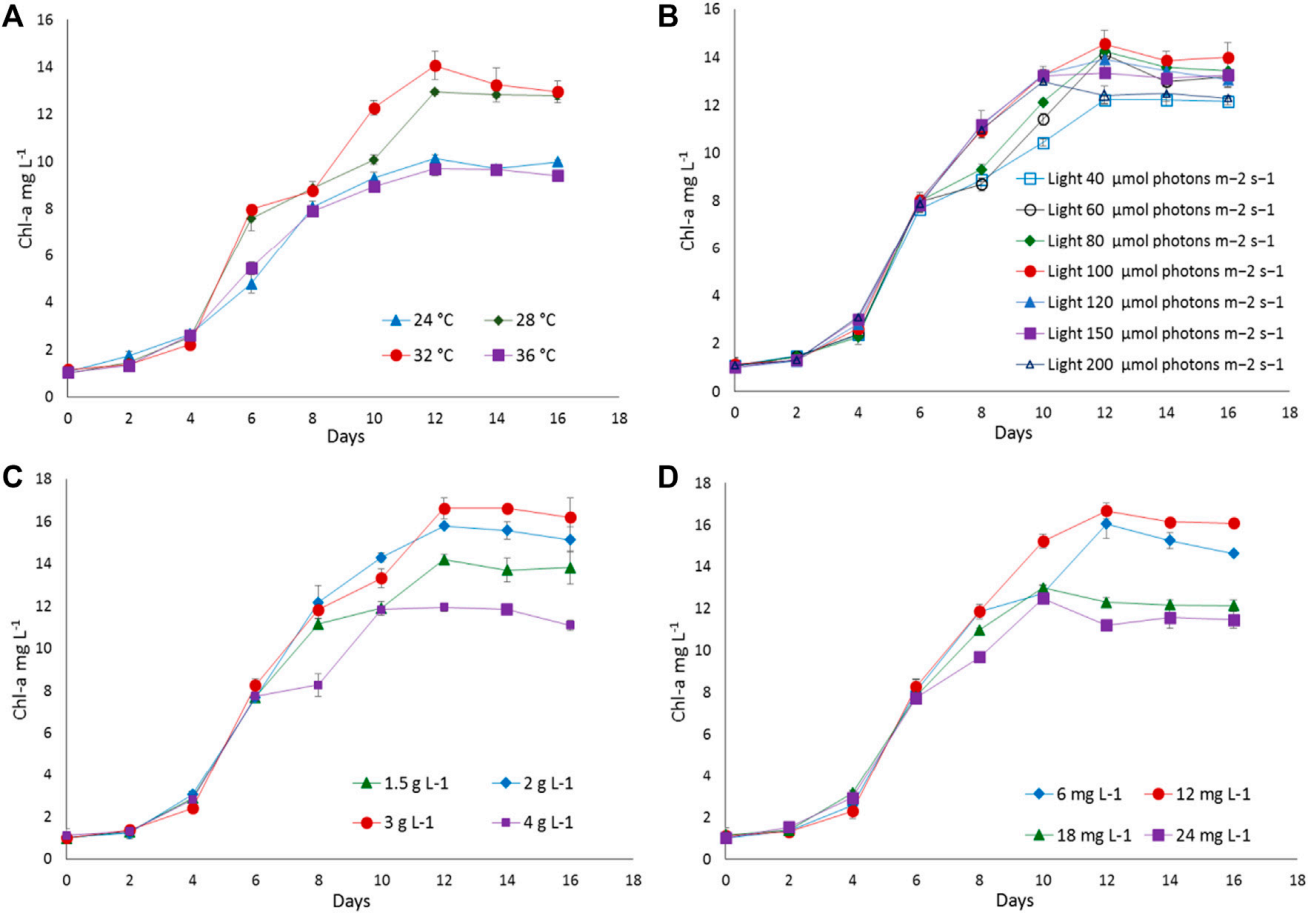
The growth response of *Desertifilum* sp. to different **(A)** temperatures, **(B)** light intensities, **(C)** sodium nitrate concentrations, and **(D)** ferric ammonium citrate concentrations. Error bars represent ±SD (n = 3).

**FIGURE 4:**
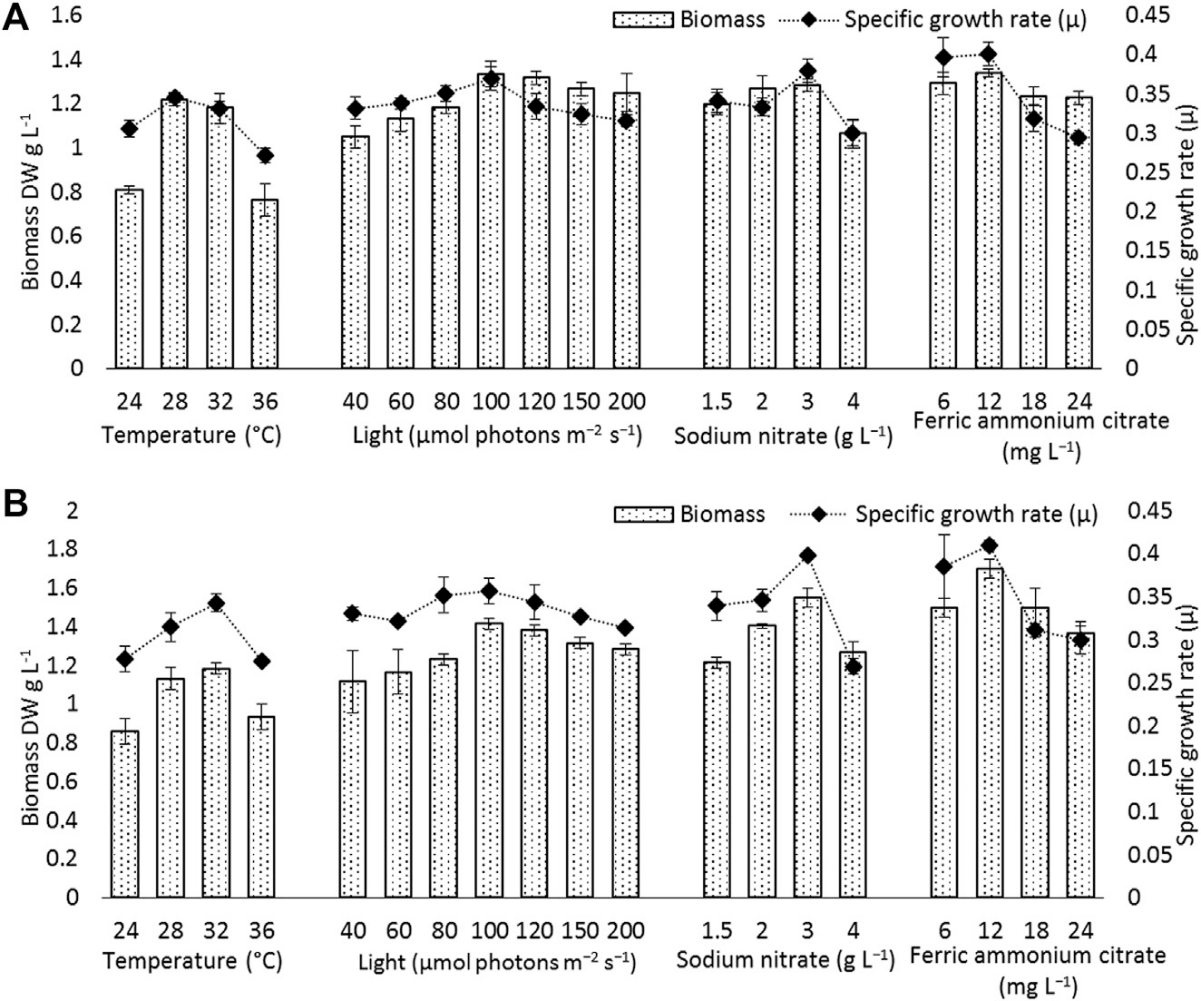
Biomass and specific growth rate of **(A)** *Euryhalinema* sp., **(B)** *Desertifilum* sp. grown under different temperature, light, and nutrient conditions (sodium nitrate and ferric ammonium citrate) at late exponential phase (12th day cultures). Error bars represent ±SD (n = 3).

**FIGURE 5:**
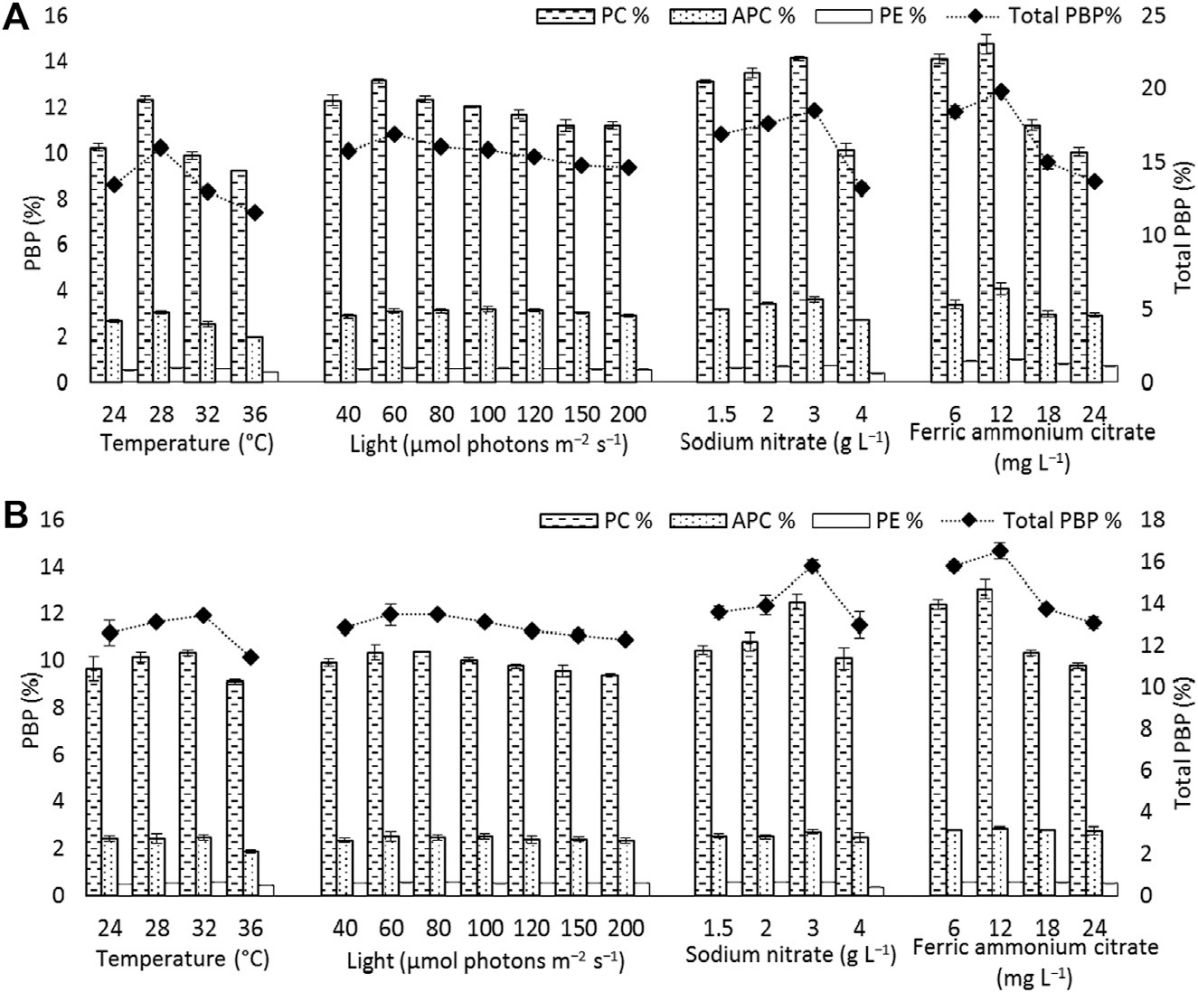
Phycobiliprotein content [phycocyanin (PC), allophycocyanin (APC), and phycoerythrin (PE)] and total phycobiliprotein (PBP) content of **(A)** *Euryhalinema* sp. **(B)** *Desertifilum* sp. grown under different temperature, light, and nutrient conditions (sodium nitrate and ferric ammonium citrate) at late exponential phase (12th day cultures). Error bars represent ±SD (n = 3).

To find out the optimum culture conditions, the effect of light and temperature on growth rate, biomass concentration, and C-PC content of two isolated strains were examined in batch cultures. Both strains grew over the temperature range of 24°C–36°C; however, the growth rates, DCW, C-PC, and Chl-a varied with the temperature. Chl-a is an effective indicator of the growth status of cyanobacteria as it reflects the state of the photosynthetic apparatus. In the present study, the time course profiles of the Chl-a contents during growth under the different temperature, light, nitrogen, and iron conditions were monitored and are depicted in Figures 2, 3, while, the biomass concentrations, growth rates, and PBP content are illustrated in Figures 4, 5.

The strain *Euryhalinema* sp. showed a maximal growth rate of 0.345 ± 0.00 days^−1^ at 28°C and reached highest DCW, Chl-a, and C-PC content of 1.21 ± 02 g L^−1^, 12.64 ± 0.24 mg L^−1^,^,^ and 12.34 ± 0.1% of dry weight, respectively. Therefore, the temperature of 28°C was found to be optimal for *Euryhalinema* sp. The DCW of *Euryhalinema* sp. grown at 24°C, 28°C, and 36°C varied significantly (*p* < 0.05). However, there is no significant difference (*p* > 0.05) between the DCW grown at 28°C and 32°C, while the *Desertifilum* sp. showed the most rapid growth rate of 0.342 ± 0.01 days^−1^ with the maximum DCW of 1.18 ± 0.02 g L^−1^, Chl-a of 14.06 ± 0.6 mg L^−1^ and C-PC content of 10.34 ± 0.10% of dry weight at 32°C. Similar to *Euryhalinema* sp., there is no significant difference (*p* > 0.05) between the DCW of *Desertifilum* sp. grown at 28°C and 32°C. The data are summarized in Supplementary Table S1.

In light intensity trails, the DCW of both *Euryhalinema* and *Desertifilum* increased significantly when the light intensity was increased from 40 to 100 μmol photons m^−2^ s^−1^. However, there is no significant difference when light intensity was further increased up to 200 μmol photons m^−2^ s^−1^. The light intensity of 100 μmol photons m^−2^ s^−1^ was found to be optimal for the growth of *Euryhalinema* and *Desertifilum* sp. with the highest specific growth rate of 0.369 ± 0.01 and 0.356 ± 0.01 days^−1^, respectively. Conversely, the C-PC content in both strains showed a decreasing trend with increasing light intensity. The maximum C-PC content of 13.14 ± 0.1% of dry weight was observed in *Euryhalinema* sp. at a light intensity of 60 μmol photons m^−2^ s^−1^, whereas, in the case of *Desertifilum* sp., the maximum C-PC content of 10.40 ± 0.1% of dry weight was obtained when using a light intensity of 80 μmol photons m^−2^ s^−1^. Since the highest C-PC content was found at lower light intensities, further experiments exploring the effects of nitrogen and iron concentrations on the growth and C-PC production were conducted at the light intensities of 60 μmol photons m^−2^ s^−1^ (*Euryhalinema* sp.) and 80 μmol photons m^−2^ s^−1^ (*Desertifilum* sp.).

### Effect of Nitrogen and Iron

To examine the effect of nitrogen source concentration on cell growth and C-PC accumulation of isolated strains, BG11 media containing 2, 3, and 4 g L^−1^ NaNO_3_ were tested whereas, the original concentration of 1.5 g L^−1^ of NaNO_3_ in BG11 media was used as a control. The cell growth rate, DCW, and C-PC content obtained with different NaNO_3_ concentrations are shown in Figures 2–5. The cell growth, DCW, and C-PC content of both strains increased when the NaNO_3_ concentration increased from 1.5 to 3 g L^−1^. However, cell growth was negatively impacted when NaNO_3_ concentration further increased from 3 to 4 g L^−1^. In both *Euryhalinema* and *Desertifilum* sp., the maximum growth rate, DCW, and C-PC content reached the highest values at 3 g L^−1^ of NaNO_3_ in the media. The values for *Euryhalinema* sp. were 0.379 ± 0.01 days^−1^, 1.28 ± 0.02 g L^−1^, and 14.15 ± 0.09% of dry weight, respectively, whereas, the values for *Desertifilum* sp. were 0.397 ± 0.00 days^−1^, 1.55 ± 0.05 g L^−1^, and 12.50 ± 0.33% of dry weight, respectively.

The effect of various iron concentrations on both strains are depicted in Figures 2–5. In both *Euryhalinema* and *Desertifilum* sp. Strains, the growth rate, DCW, and C-PC content increased with increasing ferric ammonium citrate concentration up to 12 mg L^−1^. The maximal growth rate (0.400 ± 0.01 and 0.409 ± 0.00 days^−1^), DCW (1.34 ± 0.01 and 1.7 ± 0.05 g L^−1^), and C-PC content (14.73 ± 0.39 and 13.06 ± 0.37% of dry weight) were achieved with ferric ammonium citrate concentration of 12 mg L^−1^. A further increase in ferric ammonium citrate concentration did not improve the growth and C-PC content. Therefore, the growth and C-PC content of both strains were limited by iron when the ferric ammonium citrate concentration was below 12 mg L^−1^.

Total phycobiliprotein content of *Euryhalinema* sp. and *Desertifilum* sp. was 16.02 ± 0.16% and 13.4 ± 0.01%, respectively, in normal BG-11 medium, while in enriched media, containing double strength ferric ammonium citrate and sodium nitrate, the content was increased to 19.81 ± 0.12% and 16.52 ± 0.38% for *Euryhalinema* sp. and *Desertifilum* sp., respectively (Figure 5).

### Efficiency of Cell Disruption Methods and Extraction Solution

In this study, 3 cell disruption methods with wet, oven-dried, and lyophilized biomass were evaluated to understand the efficiency of cell disruption methods for C-PC extraction from both strains using three different extraction solutions (CaCl_2_, Na-phosphate, and NH_4_Cl). The effect of various cell disruption methods on C-PC extraction from isolates were studied by quantifying C-PC content released. Table 1 shows the yield of C-PC and extracted as a function of cell disruption methods. The C-PC concentrations differed greatly by different extraction methods and solutions. Among the different cell disruption methods and extraction solutions evaluated in this investigation, freeze and thaw (−20°C, 2 h, one cycle), together with ultrasonication (2 min) from wet biomass of both strains using Na-phosphate buffer as an extraction solution, was proven to be effective for C-PC extraction, and a maximum C-PC yield of 15.02 ± 0.26% dry weight (*Euryhalinema* sp.) and 13.83 ± 0.18% dry weight (*Desertifilum* sp.) with food grade purity (purity index greater than 0.7) were observed. The extraction yield of C-PC from wet biomass is significantly higher (*p* < 0.05) than that with lyophilized and oven-dried biomass of both strains (Table 1), whereas freeze–thaw at −20°C and ultrasonication alone using Na-phosphate buffer as an extraction solution yielded lower C-PC content. Based on the C-PC yield, the extraction efficiency of cell disruption methods was ordered as freeze–thaw plus ultrasonication > ultrasonication (2 min) > freeze–thaw (−20°C).

**TABLE 1:** Cyanobacterial phycocyanin (C-PC) yield in crude extracts of *Euryhalinema* sp. and *Desertifilum* sp. obtained by different cell disruption methods and buffers (mean ± SD of the three replications).

Biomass	Extraction solution	Ultrasonication		Freeze and thaw		Ultrasonication + freeze and thaw	
		C-PC % of dry biomass					
		*Euryhalinema* sp	*Desertifilum* sp	*Euryhalinema* sp	*Desertifilum* sp	*Euryhalinema* sp	*Desertifilum* sp
Lyophilized	CaCl_2_	11.66 ± 0.37^a^	9.89 ± 0.37^d^	11.03 ± 0.2^a^	9.10 ± 0.13^f^	11.95 ± 0.24^a^	10.12 ± 0.12^d^
	NH_4_Cl	12.49 ± 0.20^b^	11.95 ± 0.44^a^	12.13 ± 0.08^b^	10.67 ± 0.44^d^	12.72 ± 0.13^b^	11.81 ± 0.15^a^
	Na-phosphate buffer	13.50 ± 0.15^c^	12.90 ± 0.47^b^	13.27 ± 0.11^c^	11.80 ± 0.13^a^	13.76 ± 0.14^c^	13.15 ± 0.38^c^
Oven dried	CaCl_2_	10.61 ± 0.26^d^	9.87 ± 0.49^d^	10.39 ± 0.26^d^	9.17 ± 0.13^f^	10.79 ± 0.24^d^	10.32 ± 0.41^d^
	NH_4_Cl	12.14 ± 0.25^b^	11.42 ± 0.26^a^	11.37 ± 0.46^a^	10.31 ± 0.21^d^	12.20 ± 0.05^b^	12.08 ± 0.19^b^
	Na-phosphate buffer	12.53 ± 0.10^b^	12.02 ± 0.32^b^	12.30 ± 0.16^b^	11.62 ± 0.16^a^	12.84 ± 0.08^b^	12.60 ± 0.14^b^
Wet	CaCl_2_	11.98 ± 0.14^a^	9.97 ± 0.13^d^	10.93 ± 0.27^d^	9.88 ± 0.06^d^	12.59 ± 0.30^b^	10.36 ± 0.49^d^
	NH_4_Cl	13.23 ± 0.16^c^	11.68 ± 0.21^a^	12.51 ± 0.24^b^	11.42 ± 0.23^a^	13.56 ± 0.38^c^	12.41 ± 0.25^b^
	Na-phosphate buffer	14.14 ± 0.27^e^	13.03 ± 0.10^c^	13.26 ± 0.15^c^	12.16 ± 0.15^b^	15.02 ± 0.26^g**^	13.83 ± 0.18^c**^

Note. Values are given as means ± SD for three replicates. Mean values followed by the same letter(s) in each column indicate that there is no significant difference (*p* > 0.05) between the results. (**) maximum C-PC yield.

### Purification and Molecular Weight Confirmation

The C-PC from the crude extracts of both strains obtained with a combined method of freeze–thaw plus ultrasonication using Na-phosphate buffer was purified by a three-step sequential method including fractional (NH₄)₂SO₄ precipitation, Sephadex G-25 gel filtration and DEAE-Sephadex ion exchange column chromatography. The data of C-PC purification from both strains are summarized in Table 2. First, both (*Euryhalinema* sp. and *Desertifilum* sp.) crude extracts were fractionated by precipitation with (NH₄)₂SO₄ at 30% and then at 70% saturation. The obtained precipitates of both crude extracts were dialyzed and further purified by passing through Sephadex G-25 media, which resulted in increased purity ratio greater than 2.5 with a recovery rate of greater than 60%, followed by purification with DEAE-Sephadex. The attained purity of *Euryhalinema* sp. and *Desertifilum* sp. C-PC fractions were 4.1 and 4.3, respectively. The final recovery rate obtained from both crude extracts after DEAE-Sephadex was 52 and 49.5%.

**TABLE 2:** Purification profile of cyanobacterial phycocyanin (C-PC) purified from the crude extract of *Euryhalinema* sp. and *Desertifilum* sp.

	Purity (A_620_/A_280_)		C-PC content (mg)		C-PC % recovery	
Purifications step	*Euryhalinema* sp	*Desertifilum* sp	*Euryhalinema* sp	*Desertifilum* sp	*Euryhalinema* sp	*Desertifilum* sp
Crude extract	0.76	0.82	149.5	140.2	100	100
Ammonium sulfate precipitation	2.1	2.2	121.8	115.3	81.5	82.3
Sephadex-G25	2.68	2.9	97.92	87.34	65.5	62.3
DEAE-Sephadex	4.1	4.3	77.74	69.39	52	49.5

**FIGURE 6:**
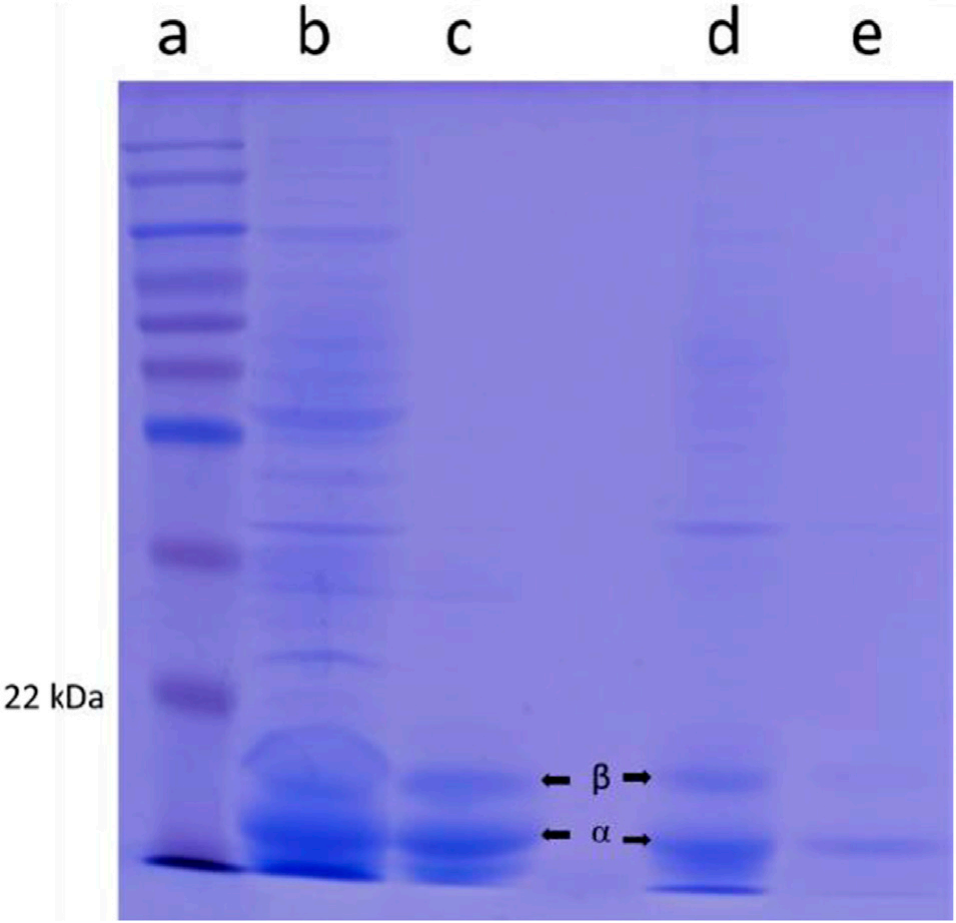
SDS-PAGE from left to right lane. **(A)** Ladder. **(B)** *Euryhalinema* sp. crude cyanobacterial phycocyanin (C-PC) extract. **(C)** *Euryhalinema* sp. purified C-PC. **(D)** *Desertifilum* sp. crude C-PC extract. **(E)** *Desertifilum* sp. purified C-PC.

The purity of the crude extracts and samples obtained after ion exchange chromatography were analyzed by SDS-PAGE. As shown in Figure 6, two bands, in Lanes B and C corresponding to the molecular masses of 15.5 and 17 kDa, represent α and β subunits of *Euryhalinema* sp. C-PC, respectively, whereas, in Lanes D and E, the two bands corresponding to the molecular masses of 15 and 17.5 kDa represent α and β subunits of *Desertifilum* sp. C-PC, respectively. According to these results, the molecular weight of monomeric subunit of both strains C-PC was estimated as 32.5 kDa.

## Discussion

One cyanobacteria isolate was identified as *Euryhalinema* based on the 16S rRNA gene sequencing. Previously, Chakraborty et al. (2019) separated the cyanobacteria strain from closely related members on the basis of polyphasic approach to taxonomic analyses encompassing molecular phylogenetic relationships of strains and designated it as a separate novel genus named *Euryhalinema*. To the best of our knowledge, no other cyanobacteria strain of this genus has been reported so far in the literature. The second cyanobacteria isolate showed resemblance with the Oscillatoriaceae family on the basis of its morphological characters, which was further confirmed as *Desertifilum* sp. by 16S rRNA gene sequencing. *Desertifilum* is a filamentous cyanobacterium of crusts and biofilms, previously described from the extreme hot and dry Thar Desert in India and warm spring in East Africa (Dadheech et al., 2014). In present study, *Desertifilum* sp. is isolated from the dynamic and harsh environment of mangroves especially high saline conditions and explored for C-PC production.

At the initial phase of our study, including the optimization of culture conditions, the growth rates of cyanobacteria cultures were monitored using Chl-a concentration. The linear relationship between chlorophyll accumulation and DCW has been previously found for other species of filamentous cyanobacteria. Therefore, chlorophyll content can be used as a proxy measure for biomass for specific culture conditions (Li et al., 2014). Both for *Euryhalinema* and *Desertifilum*, Chl-a accumulation correlated with DCW in the late exponential phase (12-day-old cultures), as was previously shown for other cyanobacteria (Kłodawska et al., 2019). Light is one of the most vital abiotic factors, which plays an important role in promoting cell growth and pigment accumulation in cyanobacteria. Usually, under extreme environmental conditions, considerable decrease in cell photosynthetic ability is observed in cyanobacteria. Both low and high light intensities cause unfavorable growth and adverse effects in cyanobacteria cells. Increase in light intensity results in increasing cell growth until a saturation point is reached, and further increase in light intensities after the saturation point can cause inhibition of the electron transfer activity of PSII (photo-inhibition) and photo oxidation that affect growth and pigment accumulation (Murata et al., 2007). Finding the ideal range of light intensity helps in attaining the optimal growth and pigment accumulation. Many researchers reported the utilization of light intensity to attain higher growth and pigment production (Takano et al., 1995; Chaneva et al., 2007; Chen et al., 2010). However, it varies with cyanobacteria species and strains. In the present investigation, the growth rate of both strains increased when the light intensity was increased from 40 to 100 μmol photons m^−2^ s^−1^. However, further increase in light intensity did not increase the growth rate. Hence, for those two species, a light intensity of 100 μmol photons m^−2^ s^−1^ was considered as optimal for their growth. It has been reported that most fresh water cyanobacteria are very sensitive to high light intensities, and they prefer low light intensities due to their low specific maintenance energy rate and pigment composition (Chaneva et al., 2007).

In addition, the C-PC content in both strains was also influenced by the light intensity and changed considerably. According to our results, the low light intensity significantly increased the C-PC accumulation, and highest C-PC content was found at a light intensity of 60 μmol photons m^−2^ s^−1^ (*Euryhalinema* sp.) and 80 μmol photons m^−2^ s^−1^ (*Desertifilum* sp.), while with increases in light intensity, the C-PC content decreased. This may be because low light intensity induce synthesis of more photosynthetic units to support light harvesting, while, high light intensity inhibits their synthesis to prevent photo damage (Murata et al., 2007). Similar to present findings, Ma*,* et al. (Ma et al., 2015) reported that a light intensity of 90 μmol photons m^−2^ s^−1^ is optimal for cell growth and PBP accumulation in *N. sphaeroides*. Likewise, Khajepour*,* et al. (Khajepour et al., 2015) reported that the PBP fraction in *Nostoc calcicola* increased under a low light intensity of 21 and 42 μmol photons m^−2^ s^−1^, whereas when increasing light intensity to 63 μmol photons m^−2^ s^−1^, the PBP content decreased. Johnson*,* et al. (Johnson et al., 2014) also found that low light intensity significantly increased the PBP content in *Nostoc* sp.

Temperature is also one of the key factors that directly affects growth and metabolic activities of cyanobacteria cells. Appropriate temperature is very important for the growth and pigment accumulation in cyanobacteria (Dadheech et al., 2014). Both lower and higher temperatures adversely affect the cellular metabolic activities, biochemical composition, and PBP synthesis (Kumar et al., 2011). Like many other metabolic processes, PBP production in cyanobacteria is also regulated by temperature, and the optimum temperature for maximum PBPs production is strain specific (Johnson et al., 2014). Data from the current study showed that temperatures of 28°C (*Euryhalinema* sp.) and 32°C (*Desertifilum* sp.) were found to be most suitable for the growth and PBP accumulation. According to the literature, optimum temperature range from 25°C to 36°C was found as optimal for PBP production in cyanobacteria (Chaneva et al., 2007; Fatma, 2009).

Nitrogen is among the most important nutrients that are required for the growth and biosynthesis of cellular components. The nitrogen concentration in culture medium greatly affects both cell growth rate and biochemical compositions of cyanobacteria. Investigators observed that nitrogen depletion in the culture medium could lead to a reduced production of photosynthetic pigments such as PBPs, while with the increase in nitrogen concentration in the medium, PBP content in cyanobacteria increases to a certain extent, and further increase does not affect its production; instead an excess of nitrate could lead to PBP inhibition (Boussiba and Richmond, 1980; Chen et al., 2013; Simeunović et al., 2013; Manirafasha et al., 2017; Khazi et al., 2018). Hence, the availability of adequate nitrogen is highly essential for C-PC production in cyanobacteria. In the present study, increasing nitrogen concentration increased biomass and C-PC production in both (*Euryhalinema* sp. and *Desertifilum* sp.) strains. The maximum biomass (1.28 ± 0.02 and 1.55 ± 0.05 g L^−1^) and C-PC (14.15 ± 0.97 and 12.50 ± 0.33% of dry biomass) was achieved with double concentration (3 g L^−1^) of NaNO_3_ in original BG11 media; further increases in NaNO_3_ to 4 g L^−1^ caused a significant decrease in biomass (1.06 ± 0.05 and 1.26 ± 0.05) and C-PC content (10.14 ± 0.26 and 10.09 ± 0.45% of dry biomass), respectively. Thus, the data showed that sufficient amount of nitrogen is required to maintain phycocyanin levels. Similarly, Kenekar and Deodhar (Kenekar and Deodhar, 2013) reported that increasing nitrate concentration increased biomass and C-PC production of cyanobacteria *Geitlerinema sulphureum* and achieved maximum biomass production at the concentration of 3.5 g L^−1^, while the PC content drastically increased at the concentrations of 3.5 g L^−1^ (8.8%) and 4.5 g L^−1^ (9.96%).

Iron is required for cell growth since many iron-containing proteins are essential for the cell to catalyze biochemical reactions involved in photosynthesis respiration, nitrogen assimilation, nitrogen fixation, and for the synthesis of major photosynthetic pigments such as Chl-a and C-PC (Chong et al., 2010). Iron limitation results in a wide range of changes in cyanobacteria. For example, the accumulation of C-PC decreases due to the decline of Chl-a, reduction of iron-containing proteins, structural changes in phycobilisomes and thylakoid membranes. The decrease in photosynthetic pigments level would affect the photosynthesis by decreasing light absorption (Xing et al., 2007). Iron limitation also reduces the ability of phycobilisomes to utilize excess light energy, and results in the formation of reactive oxygen species and, consequently, oxidative stress. It has been reported that the photosynthetic pigments (Chl, PC, APC, and carotenoids) in cyanobacteria increased under iron-replete conditions and decreased under iron-limited conditions (Hardie et al., 1983; Xing et al., 2007). In the present study, increasing ferric ammonium citrate concentration from 6 to 12 mg L^−1^ increased biomass, Chl-a and C-PC content in both strains. The highest Chl-a (15.34 ± 0.29 and 16.66 ± 0.39 mg L^−1^) and C-PC (14.73 ± 0.39 and 13.06 ± 0.37% of dry biomass) was achieved with a double concentration (12 mg L^−1^) of ferric ammonium citrate in the original BG11 media. However, further increase in ferric ammonium citrate concentration from 12 to 24 mg L^−1^ caused a significant decrease in Chl-a (10.93 ± 0.34 and 11.21 ± 0.04 mg L^−1^) and C-PC content (10.06 ± 0.20 and 9.76 ± 0.11% of dry biomass). The same effect has been observed in some other cyanobacteria. Xing, Huang, Li and Liu (Xing et al., 2007) reported that the photochemical efficiency and pigment content (Chl-a and PC) of *M. aeruginosa* and *M. wesenbergii* increased under iron-replete condition, and too low or too high concentrations of iron were able to inhibit the metabolism of *Microcystis*. For that reason, the availability of adequate iron is already established as an important factor for PC production in cyanobacteria.

The C-PC extraction efficiency, quantity, and purity may vary depending on species of cyanobacteria, characteristics of biomass (wet or dry), type of buffer, type of cell disruption method and treatment time. The PBPs are intracellular proteins that requires efficient cell disruption methods for high recovery yield. Numerous methods for PBP extraction from cyanobacteria and macroalgae have been described in the literature, and the choice of the suitable technique and efficiency of PBP extraction is dependent on the rigidity and chemical composition of the cell wall (de Jesús et al., 2016; Tavanandi et al., 2018; Pan-utai and Iamtham, 2019; Saluri et al., 2019; Pereira et al., 2020). Researchers have reported the extraction efficiency of Na-phosphate buffer and the advantages of ultrasonic-assisted extraction technique compared with the conventional methods (Hadiyanto and Suttrisnorhadi, 2016). Compared with other conventional methods, ultrasonication is the least time-consuming method, and less sonication time was enough to obtain the high C-PC yield. However, the required sonication time for cell rupture depends on the characteristics of the species and the type of biomass (wet or dry). The freeze–thaw method is a simple and cost-effective approach with no requirement of a special device. However, this method is lengthy, and the optimal number of freeze–thaw cycles differs greatly depending on the species. Data from the current study showed that freeze–thaw or ultrasonication alone is not effective for improving the C-PC release from the cell, while a combined method of ultrasonication followed by one cycle of freeze thawing significantly increased the extraction efficiency. The C-PC content from wet biomass of both strains (*Euryhalinema* sp. and *Desertifilum* sp.) obtained by a combination of ultrasonication plus freeze–thaw extraction with Na-phosphate buffer is higher (15.02 ± 0.2 and 13.83 ± 0.18% of dry biomass) than that obtained from lyophilized (13.76 ± 0.1 and 13.15 ± 0.3% of dry biomass) and oven-dried biomass (12.84 ± 0.0 and 12.60 ± 0.1% of dry biomass). Ultrasonication produces cavitation effect, which, in turn, helps in the cell wall breakage allowing the solvent to penetrate into the biomass and increasing the contact surface area between the solvent and compounds, resulting in increased mass transfer of PC from cell into the solvent. In the subsequent freeze–thaw method, when cells are frozen, intracellular ice formation occurs, which causes further disruption of cell membrane during thawing (Papadaki et al., 2017; Mogany et al., 2019). In a study with biomass of *Arthospira platensis*, higher PC extraction efficiency was attained with a combined cell disruption method of ultrasonication plus freezing and thawing (Tavanandi et al., 2018). Horváth*,* et al. (Horváth et al., 2013) also demonstrated that freeze–thaw and a subsequent sonication was the most efficient extraction method for PC from cyanobacteria. In the present study, wet, oven-dried, and lyophilized biomass was tested for the extraction of C-PC. Of all three types, oven-dried biomass yielded lower C-PC content, while higher yield was observed with wet biomass. It has been reported that drying biomass using an oven resulted in 50% loss of PC (Sarada et al., 1999; Desmorieux and Hernandez, 2004). Therefore, C-PC extraction from wet biomass was more suitable than dried biomass since it avoids loss of pigment.

The application of (NH₄)₂SO₄ precipitation and chromatographic purification has been found effective for the purification of analytical-grade C-PC from both strains. The fractional (NH₄)₂SO₄ precipitation step: first with 30% saturation salted out other proteins, but the C-PC remained soluble, and further fractionation of C-PC by precipitation with (NH₄)₂SO₄ at a concentration of 70% resulted in high-purity index C-PC with the recovery rate of greater than 80%. The dialyzed C-PC fractions were further purified by using Sephadex and DEAE. The C-PC purity index obtained after Sephadex and DEAE is greater than 4.0 satisfy the standard for analytical grade. These values are in accordance with the data reported earlier for *Spirulina* sp., *Phormidium* sp., *Lyngbya* sp. (Patel et al., 2005a), *Spirulina* sp. *Phormidium* sp., and *Pseudoscillatoria* sp. (Khazi et al., 2018) and for *Phormidium ceylanicum* (Singh et al., 2009) with a purity index of 4.1. The present study as well as the literature revealed that the DEAE method is effective to obtain analytical-grade C-PC (purity index greater than 4.0). The recovery (%) of C-PC for *Euryhalinema* sp. and *Desertifilum* sp. was 52 and 49.5%, respectively. In previous studies, with similar conventional purification strategies, the recovery of C-PC from different cyanobacteria strains was reported as 14% (Kumar et al., 2014), 45.6, 35.2, and 36.8% (Patel et al., 2005b).

## Conclusion

Owing to the outstanding bioactivities, a wide range of applications, and great economic value, the demand of phycocyanin is increasing. In this study, two cyanobacteria belonging to the genera *Euryhalinema* and *Desertifilum* were first time explored for PBP production, and their potential as a good source of phycocyanin was shown. Media and culture conditions have directly affected the growth, biomass, chlorophyll concentration, and C-PC content in both strains. The optimal culture conditions for higher biomass growth and enhanced production of phycocyanin were 28°C temperature and 60 μmol photons m^−2^ s^−1^ light intensity for *Euryhalinema* sp., whereas the optimal culture conditions for higher biomass growth and enhanced production of phycocyanin were 32°C and 80 μmol photons m^−2^ s^−1^ for *Desertifilum* sp. Double strength concentration of sodium nitrate and ferric ammonium citrate in the original BG11 media were optimal for increased growth rate and enhanced accumulation of C-PC in both strains. The combination of freeze–thaw and ultrasonication extraction strategy employed here showed promising extraction efficiency for the recovery of phycocyanin compared with other methods. Further studies are required to determine the antioxidant, antimicrobial, and anticancer potential of the produced phycocyanin.

## Data Availability

The original contributions presented in the study are included in the article/Supplementary Material, further inquiries can be directed to the corresponding author.
