# Supplementary material for: Acclimation and Characterization of Marine Cyanobacterial Strains Euryhalinema and Desertifilum for C-Phycocyanin Production
Source: Front Bioeng Biotechnol. 2021 Nov 10;9:752024. doi: 10.3389/fbioe.2021.752024 (PMC8631506; doi:10.3389/fbioe.2021.752024)
Supplement: Supplementary file 1 [file Table1.DOCX]

**Supplementary Table 1:** Biomass and specific growth rate of (a) *Euryhalinema* sp. (b) *Desertiﬁlum* sp. grown under different temperature, light and nutrients conditions (sodium nitrate and ferric ammonium citrate) at late exponential phase (12-day-old culture) (Mean ± SD of the three replications)

|  | | **(a) *Euryhalinema* sp.** | | | **(b) *Desertiﬁlum* sp.** | | |
| --- | --- | --- | --- | --- | --- | --- | --- |
|  |  | **Biomass DCW (g L^−1^)** | **Chl-a**  **mg L^−1^** | **Specific growth rate (µ)** | **Biomass DCW (g L^−1^)** | **Chl-a**  **mg L^−1^** | **Specific growth rate (µ)** |
| **Temperature (**°C**)** | **24** | 0.81±0.01 | 9.84±0.36 | 0.305±0.01 | 0.86±0.06 | 10.12±0.16 | 0.277±0.01 |
|  | **28** | 1.21±0.02 | 12.64±0.24 | 0.345±0.00 | 1.13±0.05 | 12.94±0.09 | 0.314±0.01 |
|  | **32** | 1.18±0.02 | 12.14±0.13 | 0.331±0.01 | 1.18±0.02 | 14.06±0.60 | 0.342±0.01 |
|  | **34** | 0.76±0.07 | 8.93±0.18 | 0.271±0.00 | 0.93±0.06 | 9.69±0.28 | 0.275±0.00 |
| **Photon flux density (μmol photons m^−2^ s^−1^)** | **40** | 1.05±0.05 | 12.74±0.19 | 0.331±0.01 | 1.11±0.1 | 12.22±0.14 | 0.330±0.00 |
|  | **60** | 1.13±0.05 | 12.81±0.29 | 0.337±0.00 | 1.16±0.1 | 14.12±0.62 | 0.321±0.00 |
|  | **80** | 1.18±0.02 | 13.71±0.98 | 0.350±0.00 | 1.23±0.02 | 14.25±0.22 | 0.351±0.02 |
|  | **100** | 1.33±0.05 | 14.28±0.16 | 0.369±0.01 | 1.41±0.02 | 14.56±0.55 | 0.356±0.01 |
|  | **120** | 1.31±0.02 | 14.12±0.39 | 0.334±0.01 | 1.38±0.02 | 13.91±0.49 | 0.343±0.02 |
|  | **150** | 1.26±0.02 | 13.03±0.22 | 0.324±0.01 | 1.31±0.02 | 13.35±0.22 | 0.326±0.00 |
|  | **200** | 1.25±0.08 | 12.71±0.21 | 0.316±0.00 | 1.28±0.02 | 12.41±0.38 | 0.314±0.00 |
| **Sodium nitrate (g L^−1^)** | **1.5** | 1.2±0.05 | 13.18±0.48 | 0.341±0.01 | 1.21±0.02 | 14.19±0.23 | 0.339±0.01 |
|  | **2** | 1.26±0.05 | 13.21±0.35 | 0.333±0.91 | 1.40±0.01 | 15.79±0.05 | 0.345±0.01 |
|  | **3** | 1.28±0.02 | 14.73±0.57 | 0.379±0.01 | 1.55±0.05 | 16.62±0.49 | 0.397±0.00 |
|  | **4** | 1.06±0.05 | 12.34±0.27 | 0.299±0.01 | 1.26±0.05 | 11.95±0.20 | 0.269±0.00 |
| **Ferric ammonium citrate (mg L^−1^)** | **6** | 1.29±0.05 | 14.53±0.45 | 0.396±0.02 | 1.5±0.05 | 16.05±0.70 | 0.384±0.03 |
|  | **12** | 1.34±0.01 | 15.34±0.29 | 0.400±0.01 | 1.7±0.05 | 16.66±0.39 | 0.409±0.00 |
|  | **18** | 1.23±0.04 | 12.71±0.21 | 0.319±0.01 | 1.5±0.1 | 12.30±0.21 | 0.310±0.00 |
|  | **24** | 1.22±0.03 | 10.93±0.34 | 0.293±0.00 | 1.36±0.05 | 11.21±0.04 | 0.299±0.01 |
